# CART Immunotherapy: Development, Success, and Translation to Malignant Gliomas and Other Solid Tumors

**DOI:** 10.3389/fonc.2018.00453

**Published:** 2018-10-17

**Authors:** Anna C. Filley, Mario Henriquez, Mahua Dey

**Affiliations:** Department of Neurosurgery, IU Simon Cancer Center, IU School of Medicine, Indiana University Purdue University Indianapolis, Indianapolis, IN, United States

**Keywords:** glioblastoma, malignant glioma, CAR T-cells, chimeric antigen receptor, immunotherapy

## Abstract

T cell chimeric antigen receptor (CAR) technology has allowed for the introduction of a high degree of tumor selectivity into adoptive cell transfer therapies. Evolution of this technology has produced a robust antitumor immunotherapeutic strategy that has resulted in dramatic outcomes in liquid cancers. CAR-expressing T-cells (CARTs) targeting CD19 and CD20 have been successfully used in the treatment of hematologic malignancies, producing sustained tumor regressions in a majority of treated patients. These encouraging results have led to a historic and unprecedented FDA approval of CTL019, Novartis' CAR T-cell therapy for the treatment of children and young adults with relapsed or refractory B-cell acute lymphoblastic leukemia (ALL). However, the translation of this technology to solid tumors, like malignant gliomas (MG), has thus far been unsuccessful. This review provides a timely analysis of the factors leading to the success of CART immunotherapy in the setting of hematologic malignancies, barriers limiting its success in the treatment of solid tumors, and approaches to overcome these challenges and allow the application of CART immunotherapy as a treatment modality for refractory tumors, like malignant gliomas, that are in desperate need of effective therapies.

## Introduction

The field of oncology has been revolutionized by the emergence of cellular immunotherapies that harness and augment the natural capacity of the immune system to fight cancer. As this ability is often impaired in tumor-bearing patients, one promising approach is to directly bolster deficient endogenous immune responses with adoptive T-cell therapy, which involves the passive infusion of activated, *ex vivo* expanded autologous lymphocytes that have been activated against tumor-associated antigens (TAAs) (1). These final effectors of the adaptive immune system selectively identify and destroy malignant cells, leaving healthy tissues unharmed. Furthermore, the natural development of memory cells allows for the establishment of long-lasting antitumor immunity and protection from tumor recurrence. However, as the majority of TAAs are poorly immunogenic, it is often difficult to culture a population of lymphocytes whose T-cell receptors (TCRs) have adequate avidity to exert sufficient cytotoxicity to produce lasting tumor eradication (2). This barrier can be overcome with the introduction of engineered surface receptors that have enhanced avidity and affinity for a given TAA. These chimeric antigen receptors (CARs) are comprised of an antibody-derived antigen recognition domain joined to an internal T-cell signaling domain and recognize their antigen targets through a mechanism distinct from classical TCRs (3). In addition to endowing T-cells with antibody-like specificity, these MHC-unrestricted receptors are compatible with patients of all HLA subtypes and can be used to identify tumor cells that have downregulated antigen processing and presentation functions as an adaptation to evade T-cell-mediated destruction (4). In this highly personalized form of immunotherapy, CAR-expressing T-cells (CARTs) combine the strengths of cellular and humoral immunity to equip a patient's immune system with an army of uniquely tumor-specific effector cells that have been functionally enhanced to have superior cytotoxicity, persistence, and antigen recognition capabilities in the face of tumor-induced immunosuppressive influences (5, 6).

Adoptive T-cell therapy with CAR-expressing T-cells has emerged as one of the most promising cancer immunotherapy modalities, demonstrating remarkable antitumor efficacy, particularly in the treatment of hematologic cancers. CARTs targeting CD19, a ubiquitously expressed B-cell surface antigen, have induced durable, sustained antitumor immune responses in patients with acute lymphoblastic leukemia (ALL), chronic lymphocytic leukemia, multiple myeloma, and treatment-refractory diffuse large B-cell lymphoma (DLBCL) (7–13). These encouraging results have prompted the recent, first of its kind, FDA approval of CTL019, Novartis' CAR T-cell therapy for children and young adults with relapsed or refractory B-cell ALL (14).

**Figure 1:**
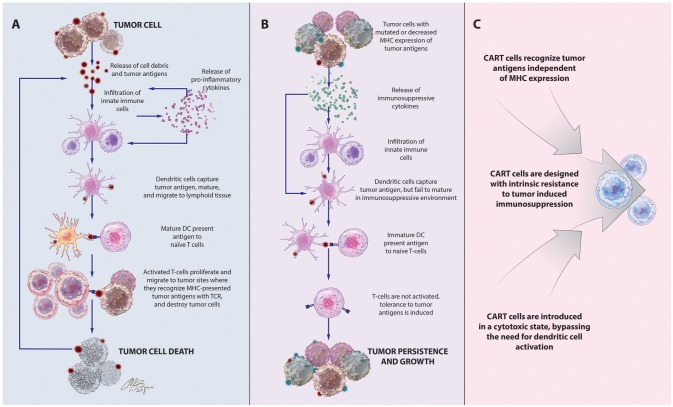
Immune-mediated interactions in solid tumors and rationale for CART immunotherapy. **(A)** Release of cell debris and tumor antigens from malignant cells activates a cascade of host antitumor immune responses, initiated by innate immune cells that release pro-inflammatory cytokines and contribute to tumor cell destruction. Among these cells are dendritic cells, which capture tumor antigens, mature in response to the pro-inflammatory cytokines in the environment, and travel to lymphoid tissues to stimulate T-cell proliferation and activation of antigen-specific adaptive immune responses leading to tumor death. **(B)**. Tumors often develop adaptations to evade detection and destruction by the host immune system. Through the recruitment of suppressive leukocytes and elaboration of immunosuppressive cytokines, tumors inhibit the function of infiltrating immune cells, including dendritic cells. Incompletely matured DCs are unable to effectively activate naïve T cells, instead inducing T-cell anergy, apoptosis, or tolerance to tumor-associated antigens. Downregulation of antigen-presenting machinery and the development of antigen-loss variants enable tumor cells to escape detection by infiltrating immune cells. **(C)** CAR T-cells, which recognize antigens via a mechanism distinct from TCR stimulation, bypass the need for DC antigen presentation and are unaffected by MHC downregulation. CAR structure and culture conditions can also be optimized to create CART populations with superior cytotoxicity and resistance to tumor-induced suppressive influences.

Inspired by this success in liquid tumors, there has been great interest in expanding the use of CART technology to the treatment of solid tumors like glioblastoma (GBM), a highly aggressive form of primary brain cancer for which there is no known cure (15). Supporting the exploration of T-cell-based therapies in solid tumors is the strong positive correlation between the degree of intratumoral infiltration with antigen-specific cytotoxic T-cells (CTLs) and overall patient survival (16, 17). Given the importance of the delicate balance between host and tumor immune responses on the ultimate course of disease, these patients are likely to benefit from highly sophisticated treatments like CART immunotherapy that can both strengthen antitumor immunity and overcome tumor-induced immunosuppressive influences, to tip the balance toward tumor cell death, Figure 1.

CART immunotherapy may also have superior therapeutic efficacy in the treatment of solid cancers that are otherwise poorly accessible to standard therapies, such as malignant gliomas (MG). Nestled within the brain parenchyma, these tumors are particularly dangerous to remove with relatively nonspecific therapies like surgery and radiation due to the risk of damaging surrounding eloquent brain tissue. Furthermore, the highly infiltrative growth pattern of aggressive tumors like GBM makes a complete, curative resection impossible. Intracranial tumors are further isolated from the systemic circulation by the presence of a blood brain barrier, which restricts the passage of most chemotherapeutic agents, preventing their therapeutic accumulation within tumor sites and increasing the risk of systemic toxicity (18). With a known ability to cross the blood brain barrier, activated T-cell-based therapies have potential to overcome these challenges to safely and effectively reach surgically inaccessible malignant cells. A cellular-based approach using functionally enhanced, antigen-specific CTLs, which have cytotoxic functions restricted to antigen-expressing tumor cells, represents a particularly promising treatment strategy for these intracranial tumors (19).

Unfortunately, in stark contrast to the success observed with hematologic malignancies, CARTs have exhibited limited efficacy in the treatment of solid tumors. However, this form of immunotherapy is still in its infancy and there have been many exciting advances in recent years with significant potential to revolutionize CART application to solid tumors. In this review, we highlight the landmark discoveries that have led to the success of CART immunotherapy in hematologic malignancies, identify barriers to its application in solid tumors, and propose research avenues for novel approaches to overcome these barriers and allow successful application of CART immunotherapy to treatment-resistant solid tumors, with a special emphasis on the primary malignant brain cancer, GBM.

## Chimeric antigen receptors

**Figure 2:**
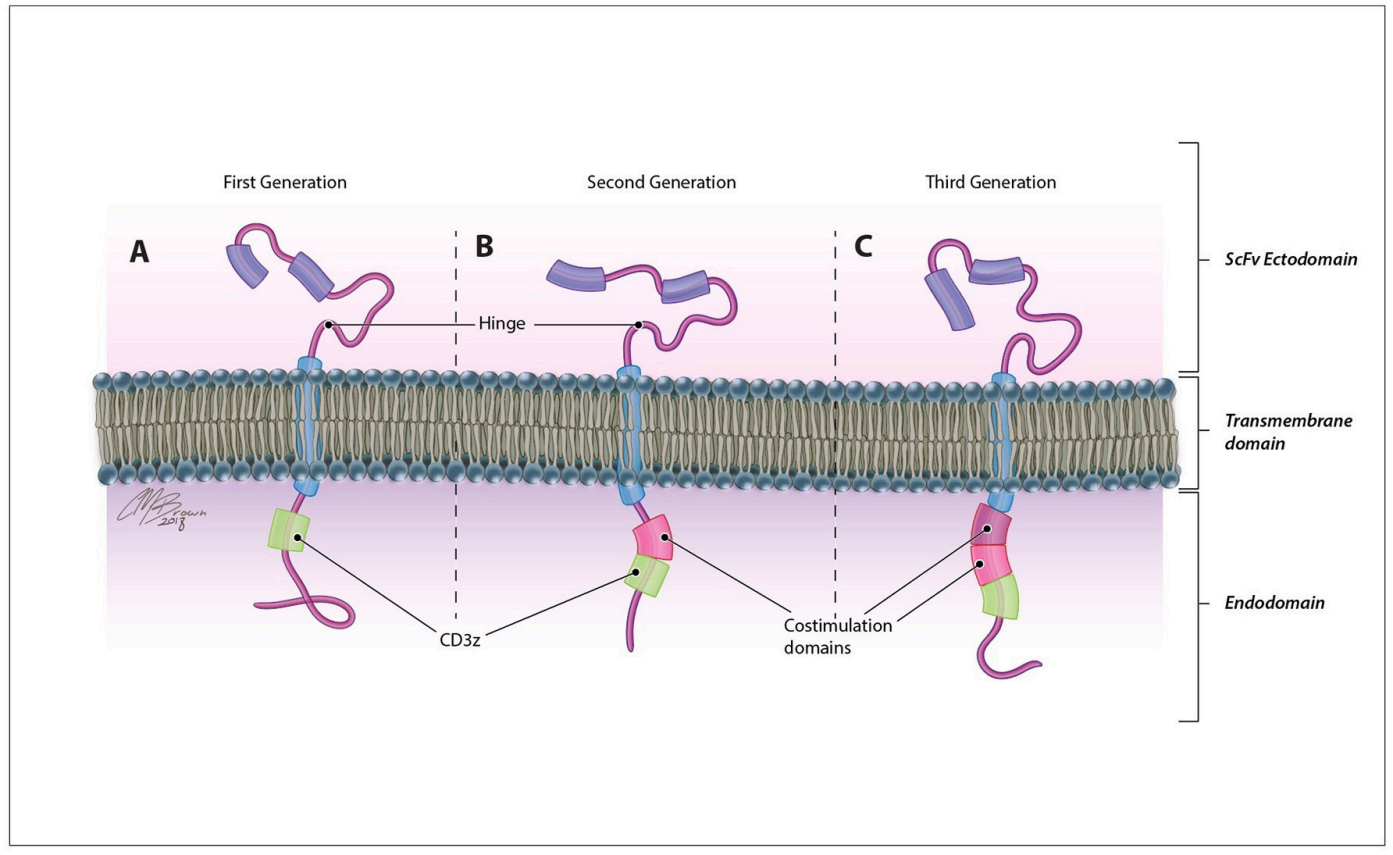
CAR structure. CARs are comprised of an antigen-recognition ectodomain derived from the single-chain variable fragment (scFv) of a monoclonal antibody connected by a flexible hinge and transmembrane segment to an intracellular endodomain. Originally derived from the CD3ζ domain of the classical TCR in **(A)** first generation CARs, this intracellular signaling component may contain **(B)** one (second generation) or **(C)** two (third generation) additional costimulatory domains that enhance the proliferation, persistence, and efficacy of adoptively transferred cells.

CARs are synthetic antigen receptors that can be introduced into an immune cell to retarget its cytotoxicity toward a specific tumor antigen with a greater degree of specificity. In contrast to traditional TCRs, which identify intracellularly derived peptide antigens presented by MHC molecules, CARs directly identify antigens expressed on the surface of tumor cells therefore are not restricted by patient HLA subtype and can recognize a variety of antigen structures including proteins, carbohydrates, and glycolipids (20). These membrane-bound fusion proteins couple a high avidity extracellular antigen recognition moiety derived from the single-chain variable fragment of a monoclonal antibody with a modified TCR intracellular signaling domain Figure 2 (3). Originally derived from the CD3ζ chain of the traditional TCR (21), CAR endodomains have undergone generational changes to include one or more costimulatory domains, most commonly CD28 and 41BB, to enhance the persistence and cytotoxicity of CAR-expressing cells (22, 23).

Following receptor design, a gene encoding the CAR construct is transfected into the genome of T-cell isolates using gene-therapy viral vectors, and the resulting CAR T-cells are expanded *ex vivo* and re-infused into the patient. Classical CART therapy is restricted to the use of autologous leukocytes due to the presence of self-identification molecules on the surface of T-cells. However, treatment-related lymphopenia may preclude the ability to isolate sufficient peripheral blood leukocytes for treatment. These patients may benefit from donor cell based therapies that use universal CAR T-cells that have been genetically modified to completely lack endogenous expression of self-identifying molecules like the TCR and HLA class I molecules (24, 25).

## CART and hematological malignancies

**Figure 3:**
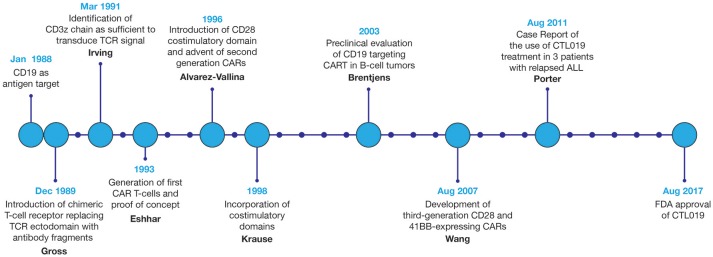
Development of CART immunotherapy. Following development of the first chimeric T-cell receptor in 1989, early preclinical studies of the first CARTs demonstrated the ability to selectively identify and destroy antigen-expressing tumor cells (5, 6). However, upon adoptive transfer into live patients, T-cells expressing these first-generation CARs displayed limited persistence and were often rendered anergic due to the absence of costimulatory signals within the tumor microenvironment (TME) (26). With the introduction of costimulatory domains to provide these necessary activating signals, CART immunotherapy experienced a dramatic improvement in therapeutic efficacy (22). Optimization of CAR structure and *ex vivo* culture conditions to improve CART persistence, cytotoxicity, and resistance to tumor-induced immunosuppression remains an area of continued research. Evaluated in a variety of tumor types, CART immunotherapy has been markedly successful in the eradication of liquid tumors, culminating in the FDA approval of CART immunotherapy for the treatment of relapsed or refractory B-cell ALL in 2017.

Since the first design of chimeric T-cell receptors in 1989, CAR technology and the field of CART immunotherapy have advanced immensely, with several key landmark achievements that have propelled this therapeutic modality to the forefront of cancer treatment, Figure 3 (3). Evaluated in a variety of tumor types, CART immunotherapy has produced particularly successful clinical responses in the treatment of hematologic malignancies. CAR T-cells targeting B-cell surface antigens, particularly CD19, have demonstrated a marked ability to eradicate liquid tumors (27, 28). Upon introduction into human clinical trials, CART immunotherapy induced sustained tumor regression in a majority of treated patients (7–13). This success prompted the recent FDA approval of CTL019, Novartis's CAR T-cell therapy for the treatment of children and young adults with relapsed or refractory B-cell ALL, bringing CART immunotherapy to the front lines of standard cancer treatment (14).

Reflecting on the success of CART immunotherapy in hematologic cancers, tumors of B-cell origin possess several qualities that have provided for this exceptional antitumor efficacy. Primarily, this can be attributed to the virtually ideal properties of CD19 as an antigen target. Ideal antigens for CAR generation are tumor exclusive, expressed by all malignant cells, and have a function crucial to tumor growth and survival. Together, these qualities maximize tumoricidal capacity, prevent immune evasion, and reduce the risk of toxicity stemming from CART destruction of antigen-expressing healthy cells. Uniformly expressed by malignant cells of B-cell cancers CD19 is lineage-restricted B-cell surface marker that is not present on other tissues of the body (29). Although non-cancerous immune cells also express CD19, transient inadvertent destruction of these populations during treatment does not result in therapy-limiting toxicity (30). Additionally, malignant cells of hematologic cancers typically reside in locations of routine T-cell migration (peripheral blood, lymph nodes, and bone marrow), making them readily accessible to systemically delivered CAR T-cells. A relatively disseminated distribution also precludes tumor isolation by physical barriers such as a dense fibrous stroma and the development of a highly immunosuppressive local tumor microenvironment (TME). The combination of these factors has allowed for the rapid, efficient, and uninhibited destruction of CD19-positive malignant cells leading to regression of B-cell cancers.

## CART and solid malignancies

CARs have also been developed against a variety of solid tumor surface antigens including mesothelin (31), carcinoembryonic antigen (CEA) (32), disialoganglioside (GD2) (33), interleukin-13 receptor α2 (IL-13Rα2) (34), mucin-1 (MUC1) (35, 36), ephrin type-A receptor 2 (EphA2), (37), human epidermal growth factor receptor 2 (HER2) (38) and other growth factor receptors. Currently there are several ongoing clinical trials assessing the safety and efficacy of CART immunotherapy in various solid malignancies (Table 1).

**Table 1:** List of CART immunotherapy clinical trials in solid tumors (except gliomas).

**Malignancy**	**Phase**	**N**	**Name of Trial**	**Therapeutic Compounds**	**Clinical Trial Identifier**	**Status**
GPC3 Positive Hepatocellular Carcinoma	1/2	60	CAR-T Cell Immunotherapy for HCC Targeting GPC3	GPC3	NCT02723942	Completed
Carcinoma, Hepatocellular	1/2	30^*^	A Study of GPC3 Redirected Autologous T Cells for Advanced HCC (GPC3-CART)	GPC3	NCT02715362	Recruiting
Advanced Lung Cancer	1	22^*^	CAR-T Cell Immunotherapy for Advanced Lung Cancer	PD-L1	NCT03330834	Not Yet Open
Advanced Solid Tumor	1/2	40^*^	CTLA-4 and PD-1 Antibodies Expressing MUC1-CAR-T Cells for MUC1 Positive Advanced Solid Tumor	MUC1	NCT03179007	Recruiting
Colon Cancer, Esophageal Carcinoma, Pancreatic Cancer, Prostate Cancer, Gastric Cancer, Hepatic Carcinoma	1/2	60^*^	A Clinical Research of CAR T Cells Targeting EpCAM Positive Cancer (CARTEPC)	EpCAM	NCT03013712	Recruiting
Pancreatic Cancer	1	30^*^	A Study of Mesothelin Redirected Autologous T Cells for Advanced Pancreatic Carcinoma (meso-CART)	Mesothelin	NCT02706782	Recruiting
Lung Cancer	1	30^*^	PSCA/MUC1/PD-L1/CD80/86-CAR-T Cells Immunotherapy Against Cancers	PSCA, MUC1, PD-L1 or CD80/86	NCT03198052	Recruiting
Sarcoma, Osteoid Sarcoma, Ewing Sarcoma	1/2	20^*^	Safety and Efficacy Evaluation of 4th Generation Safety-engineered CAR T Cells Targeting Sarcomas	Sarcoma-specific CAR-T cells	NCT03356782	Recruiting
Lung Cancer	1/2	20^*^	Intervention of CAR-T Against Lung Cancer	Lung cancer-specific CAR-T cells	NCT03356808	Not Yet Open
Breast Cancer, Ovarian Cancer, Lung Cancer, Gastric Cancer, Colorectal Cancer, Glioma, Pancreatic Cancer	1/2	60^*^	A Clinical Research of CAR T Cells Targeting HER2 Positive Cancer	HER2	NCT02713984	Recruiting
Cervical Cancer	1/2	20^*^	Intervention of CAR-T Against Cervical Cancer	Cervical cancer-specific CAR-T cells	NCT03356795	Recruiting
Hepatocellular Carcinoma, Squamous Cell Lung Cancer	1	30^*^	GPC3-T2-CAR-T Cells for Immunotherapy of Cancer With GPC3 Expression	GPC3	NCT03198546	Recruiting
Liver Metastases	1/2	20^*^	A Study of MG7 Redirected Autologous T Cells for Advanced MG7 Positive Liver Metastases(MG7-CART)	MG7	NCT02862704	Recruiting
EGFR-positive Colorectal Cancer	1/2	20^*^	EGFR CART Cells for Patients With Metastatic Colorectal Cancer	EGFR	NCT03152435	Recruiting
Advanced Solid Tumor	1/2	40^*^	CTLA-4 and PD-1 Antibodies Expressing EGFR-CAR-T Cells for EGFR Positive Advanced Solid Tumor	EGFR	NCT03182816	Recruiting
Liver Neoplasms	2	25^*^	Study Evaluating the Efficacy and Safety With CAR-T for Liver Cancer (EECLC)	EPCAM	NCT02729493	Recruiting
Stomach Neoplasms	2	19^*^	Study Evaluating the Efficacy and Safety With CAR-T for Stomach Cancer (EECSC)	EPCAM	NCT02725125	Recruiting
Liver Metastases	1	5^*^	CAR-T Hepatic Artery Infusions for CEA-Expressing Liver Metastases (HITM-SURE)	CEA	NCT02850536	Recruiting
Carcinoma, Hepatocellular	1/2	10^*^	A Study of GPC3-targeted T Cells by Intratumor Injection for Advanced HCC (GPC3-CART)	GPC3	NCT03130712	Recruiting
Liver Metastases	1	8	CAR-T Hepatic Artery Infusions and Sir-Spheres for Liver Metastases (HITM-SIR)	CEA	NCT02416466	Ongoing, not recruiting
Advanced EGFR-positive Solid Tumors	1/2	60^*^	Treatment of Chemotherapy Refractory EGFR (Epidermal Growth Factor Receptor) Positive Advanced Solid Tumors (CART-EGFR) (CART-EGFR)	EGFR	NCT01869166	Complete, status unknown
Hepatocellular Carcinoma, Liver Cancer, Liver Neoplasms, Metastatic Liver Cancer	1	18^*^	Clinical Study of ET1402L1-CAR T Cells in AFP Expressing Hepatocellular Carcinoma	ET1402L1	NCT03349255	Recruiting
Malignant Neoplasm of Nasopharynx TNM Staging Distant Metastasis (M), Breast Cancer Recurrent	1	30^*^	EpCAM CAR-T for Treatment of Nasopharyngeal Carcinoma and Breast Cancer	EpCAM	NCT02915445	Recruiting
Mesothelin Positive Tumors	1	20^*^	Anti-mesothelin CAR T Cells for Patients With Recurrent or Metastatic Malignant Tumors	Mesothelin	NCT02930993	Recruiting
Hepatocellular Carcinoma	1	13	Anti-GPC3 CAR T for Treating Patients With Advanced HCC	GPC3	NCT02395250	Terminated
Hepatocellular Carcinoma, Non-small Cell Lung Cancer, Pancreatic Carcinoma, Triple-Negative Invasive Breast Carcinoma	1/2	20^*^	Phase I/II Study of Anti-Mucin1 (MUC1) CAR T Cells for Patients With MUC1+ Advanced Refractory Solid Tumor	MUC1	NCT02587689	Recruiting
Pancreatic Cancer	1	4	Pilot Study of Autologous T-cells in Patients With Metastatic Pancreatic Cancer	Mesothelin	NCT02465983	Completed
Advanced HER-2 Positive Solid Tumors	1/2	10^*^	Treatment of Chemotherapy Refractory Human Epidermal growth Factor Receptor-2(HER-2) Positive Advanced Solid Tumors (CART-HER-2)	HER-2	NCT01935843	Recruiting
Lung Cancer, Colorectal Cancer, Gastric Cancer, Breast Cancer, Pancreatic Cancer	1	75^*^	A Clinical Research of CAR T Cells Targeting CEA Positive Cancer	CEA	NCT02349724	Recruiting
Adult Advanced Cancer, Solid Tumor	1/2	40^*^	PD-1 Antibody Expressing CAR T Cells for Mesothelin Positive Advanced Malignancies	Mesothelin	NCT03030001	Recruiting
Breast Cancer	1/2	60	Chimeric Antigen Receptor-Modified T Cells for Breast Cancer	HER-2	NCT02547961	Completed
Pancreatic Cancer	Early 1	10^*^	Evaluate the Safety and Efficacy of CAR-T in the Treatment of Pancreatic Cancer.	Mesothelin, PSCA, CEA, HER2, MUC1, EGFRvIII	NCT03267173	Recruiting
Prostate Cancer	1	18^*^	CART-PSMA-TGFβRDN Cells for Castrate-Resistant Prostate Cancer	PSMA, TGFβRDN	NCT03089203	Recruiting
Carcinoma, Hepatocellular, Pancreatic Cancer Metastatic, Colorectal Cancer Metastatic	1/2	20^*^	A Study of Chimeric Antigen Receptor T Cells Combined With Interventional Therapy in Advanced Liver Malignancy	GPC3, Mesothelin, or CEA	NCT02959151	Recruiting
Advanced Solid Tumor	1/2	40^*^	CTLA-4 and PD-1 Antibodies Expressing Mesothelin-CAR-T Cells for Mesothelin Positive Advanced Solid Tumor	Mesothelin	NCT03182803	Recruiting
Metastatic Pancreatic (Ductal) Adenocarcinoma, Epithelial Ovarian Cancer, Malignant Epithelial Pleural Mesothelioma	1	19	CART-meso in Mesothelin Expressing Cancers	Mesothelin	NCT02159716	Completed
Nasopharyngeal Neoplasms	1/2	20^*^	A New EBV Related Technologies of T Cells in Treating Malignant Tumors and Clinical Application	LMP1	NCT02980315	Recruiting
Advanced Malignancies	1/2	20^*^	PD-1 Antibody Expressing CAR-T Cells for EGFR Family Member Positive Advanced Solid Tumor	EGFR	NCT02873390	Recruiting
Hepatocellular Carcinoma	1/2	20^*^	Anti-GPC3 CAR-T for Treating GPC3-positive Advanced Hepatocellular Carcinoma (HCC)	GPC3	NCT03084380	Recruiting
Advanced Solid Tumor	1/2	20^*^	PD-1 Antibody Expressing CAR-T Cells for EGFR Family Member Positive Advanced Solid Tumor (Lung, Liver and Stomach)	EGFR	NCT02862028	Recruiting
Pancreatic Cancer	1	18^*^	CAR T Cell Immunotherapy for Pancreatic Cancer	Mesothelin	NCT03323944	Recruiting
Lung Adenocarcinoma, Ovarian Cancer, Peritoneal Carcinoma, Fallopian Tube Cancer, Mesotheliomas Pleural, Mesothelioma Peritoneum	1	30^*^	CAR T Cells in Mesothelin Expressing Cancers	Mesothelin	NCT03054298	Ongoing, not recruiting
Bladder Cancer, Urothelial Carcinoma Bladder	1/2	20^*^	Intervention of Bladder Cancer by CAR-T	PSMA and FRa	NCT03185468	Recruiting
Malignant Mesothelioma, Pancreatic Cancer, Ovarian Tumor, Triple Negative Breast Cancer, Endometrial Cancer, Other Mesothelin Positive Tumors	1	20^*^	Treatment of Relapsed and/or Chemotherapy Refractory Advanced Malignancies by CART-meso	Mesothelin	NCT02580747	Recruiting
Liver Cancer, Pancreatic Cancer, Brain Tumor, Breast Cancer, Ovarian Tumor, Colorectal Cancer, Acute Myeloid and Lymphoid Leukemias	1	20^*^	Treatment of Relapsed and/or Chemotherapy Refractory Advanced Malignancies by CART133	CD133	NCT02541370	Recruiting
Lung Squamous Cell Carcinoma	1	20^*^	Anti-GPC3 CAR T for Recurrent or Refractory Lung Squamous Cell Carcinoma	GPC3	NCT02876978	Recruiting
Malignant Melanoma, Breast Cancer	Early 1	10^*^	Autologous T Cells Expressing MET scFv CAR (RNA CART-cMET)	cMET	NCT03060356	Recruiting
Sarcoma, Osteosarcoma, Neuroblastoma, Melanoma	1	15	A Phase I Trial of T Cells Expressing an Anti-GD2 Chimeric Antigen Receptor in Children and Young Adults With GD2+ Solid Tumors	GD2	NCT02107963	Completed
Non-Resectable Pancreatic Cancer	1	30^*^	Prostate Stem Cell Antigen (PSCA)-Specific CAR T Cells In Subjects With Non-Resectable Pancreatic Cancer	PSCA	NCT02744287	Recruiting
Hepatocellular Carcinoma	1	14^*^	Glypican 3-specific Chimeric Antigen Receptor Expressing T Cells for Hepatocellular Carcinoma (GLYCAR) (GLYCAR)	Glypican 3	NCT02905188	Recruiting
Metastatic Cancer, Metastatic Melanoma, Renal Cancer	1/2	24	CAR T Cell Receptor Immunotherapy Targeting VEGFR2 for Patients With Metastatic Cancer	VEGFR2	NCT01218867	Completed
Cervical Cancer, Pancreatic Cancer, Ovarian Cancer, Mesothelioma, Lung Cancer	1/2	136^*^	CAR T Cell Receptor Immunotherapy Targeting Mesothelin for Patients With Metastatic Cancer	Mesothelin	NCT01583686	Recruiting
Solid Tumor	1/2	100^*^	Study on GD2 Positive Solid Tumors by 4SCAR-GD2	GD2	NCT02992210	Recruiting
Colon Cancer Liver Metastasis	1	18^*^	Hepatic Transarterial Administrations of NKR-2 in Patients With Unresectable Liver Metastases From Colorectal Cancer (LINK)	NKR-2	NCT03370198	Recruiting
Metastatic Breast Cancer, Triple Negative Breast Cancer	1	15^*^	cMet CAR RNA T Cells Targeting Breast Cancer	cMet RNA	NCT01837602	Ongoing, not recruiting
Colorectal Cancer, Ovarian Cancer, Urothelial Carcinoma, Triple-negative Breast Cancer, Pancreatic Cancer	1	24^*^	A Dose Escalation Phase I Study to Assess the Safety and Clinical Activity of Multiple Cancer Indications (THINK)	NKR-2	NCT03018405	Recruiting
Stage IV Breast Cancer, Stage IV Non-Small Cell Lung Cancer, Triple-Negative Breast Carcinoma	1	60^*^	Genetically Modified T-Cell Therapy in Treating Patients With Advanced ROR1+ Malignancies	ROR1	NCT02706392	Recruiting
Hepatocellular Carcinoma		20^*^	CAR-GPC3 T Cells in Patients With Refractory Hepatocellular Carcinoma	GPC3	NCT03146234	Recruiting
Sarcomas	1	26^*^	iC9-GD2-CAR-VZV-CTLs/Refractory or Metastatic GD2-positive Sarcoma/VEGAS	GD2	NCT01953900	Ongoing, not recruiting
Pancreatic Cancer, Renal Cell Cancer, Breast Cancer, Melanoma, Ovarian Cancer	1/2	113^*^	Administering Peripheral Blood Lymphocytes Transduced With a CD70-Binding Chimeric Antigen Receptor to People With CD70 Expressing Cancers	hCD70	NCT02830724	Recruiting
Breast Cancer, Metastatic HER2-negative Breast Cancer	1	24^*^	T-Cell Therapy for Advanced Breast Cancer	Mesothelin	NCT02792114	Recruiting

* *Means estimated sample size*.

With respect to GBM, much of the current CAR development is focused on the following antigen targets: EGFRvIII, IL-13Rα2, and HER2. EGFRvIII is a mutated form of the epidermal growth factor receptor (EGFR), resulting from a tumor-specific in-frame deletion creating a constitutively active surface receptor protein. Present in approximately 30% of GBMs, this mutant receptor enhances glioma cell proliferation, angiogenesis, and invasiveness (39) and is independently associated with a poor prognosis (40). Preclinical studies have established the ability of T-cells targeting this unique, tumor-specific epitope to proliferate and release cytokines in response to stimulation with the mutant EGFRvIII antigen, but not wild-type EGFR (41). In preclinical studies, EGFRvIII-targeting CARTs effectively traffic to tumor sites and suppress the growth of glioma xenografts in murine models (42). In human clinical trials, preliminary reports from a phase I study in 10 patients with recurrent GBM established the safety and feasibility of EGFRvIII-targeting CART immunotherapy. Adoptively transferred cells were shown to proliferate within the peripheral blood and traffic to intracranial tumor sites, exerting antitumor effects without any evidence of cross-toxicity with wild-type EGFR. Interestingly, analysis of pre- and post-treatment tumor samples revealed post-treatment decreases in antigen expression and an increased presence of inhibitory immune checkpoint molecules and regulatory T-cell infiltrates, indicative of evasive tumor responses. The median overall survival was approximately 8 months, with one patient experiencing residual stable disease at 18 months (43). Clinical trials are currently ongoing specifically to assess efficacy.

Another encouraging target for patients with GBM is interleukin-13 receptor alpha-2 (IL-13Rα2). Not expressed by healthy tissues, IL-13Rα2 is overexpressed in nearly all GBM tumors (44). IL-13Rα2-targeting CARTs have been shown to selectively target and kill IL-13Rα2-positive tumor cells *in vitro* and *in vivo*, producing regression of xenograft glioma tumors in a murine model (34). Based on this success, several human clinical trials were initiated with IL-13Rα2-targeting CARTs. Transient anti-glioma responses, without significant associated toxicities, were observed in a subset of treated patients (45, 46).

Human epidermal growth factor receptor 2 (HER2) is a transmembrane tyrosine kinase receptor expressed on a variety of healthy tissues. Overexpressed in many solid tumors, including approximately 15% of GBM tumors, HER2 has been identified as an independent negative prognostic indicator for GBM patient survival (47). In a recent phase I dose-escalation study of HER2-specific CAR-modified virus-specific T-cells for the treatment of progressive HER2-positive glioblastoma, therapy was well tolerated without any dose-limiting toxicities. Median overall survival was 11.1 months from the time of first infusion, and 24.5 months from initial diagnosis (48).

CARTs targeting these and other TAAs have shown significant antitumor activity in *in vitro* and *in vivo* preclinical studies (Table 2), and many of these therapies have reached the stage of human clinical trials for patients with GBM (Table 3). Across all solid tumors, one of the most promising studies has occurred in the treatment of neuroblastoma with GD2-targeting CARTs, where complete remission was achieved in 3 of 11 treated patients (57). However, in stark contrast to the efficacy in hematologic malignancies, no CAR T-cell therapy has been shown to induce consistent, lasting regression of solid tumor in human patients.

**Table 2:** List of antigens targeted by CART in pre-clinical glioma model.

**Antigen**	**Generation**	**Other Treatments/Modifications**	**Reference**
NKG2D	Second Generation	Radiotherapy/ NKG2D–CD3ζ-DAP10	Weiss et al. (49)
GD2	Third Generation	Engineered with active IL-7 receptor (C7R) and a 41BB.ζ signaling endodomain	Shum et al. (50)
IL13Rα2	Second Generation	Engineered contain either a CD28.ζ, 41BB.ζ CD28.OX40.ζ, or CD28.41BB.ζ endodomain	Krenciute et al. (51)
EGFRvIII	Third Generation	Engineered to express miR-17-92 with CD28, 41BB and CD3ζ signaling	Ohno et al. (52)
EGFRvIII	Third Generation	Engineered to contain a CD28-41BB-CD3ζ endodomain	Choi et al. (53)
EGFRvIII	Second Generation	Engineered with ICOS and CD3ζ signaling domain	Shen et al. (54)
NY-ESO-1	N/A	Decitabine, in culture/ modified CDR	Everson et al. (55)
EGFRvIII	Third Generation	Engineered with mouse CD8 trans-membrane and mouse CD28, 4-1BB, and CD3ζ intracellular regions	Sampson et al. (56)

**Table 3:** List of CART immunotherapy clinical trials in malignant gliomas.

**Malignancy**	**Phase**	**N**	**Name of Trial**	**Target**	**Clinical Trial Identifier**	**Status**
Advanced Glioma	1	10^*^	CAR T Cells in Treating Patients With Malignant Gliomas Overexpressing EGFR	EGFR	NCT02331693	Completed, Unknown
GD2 Positive Glioma	1/2	60	CAR-T Cell Immunotherapy for GD2 Positive Glioma Patients	GD2	NCT03252171	Completed
EphA2 Positive Malignant Glioma	1/2	60	CAR-T Cell Immunotherapy for EphA2 Positive Malignant Glioma Patients	EphA2	NCT02575261	Completed
Glioblastoma	1/2	107^*^	CAR T Cell Receptor Immunotherapy Targeting EGFRvIII for Patients With Malignant Gliomas Expressing EGFRvIII	EGFRvIII	NCT01454596	Recruiting
Malignant Glioma, Colorectal Carcinoma, Gastric Carcinoma	1/2	20^*^	CAR-T Cell Immunotherapy in MUC1 Positive Solid Tumor	MUC1	NCT02617134	Recruiting
Residual or Recurrent EGFRvIII+ Glioma	1	12^*^	Autologous T Cells Redirected to EGFRVIII-With a Chimeric Antigen Receptor in Patients With EGFRVIII+ Glioblastoma	EGFRvIII	NCT02209376	Ongoing, Not recruiting
Malignant Glioma, Refractory Brain Neoplasm, Recurrent Brain Neoplasm	1	135^*^	Genetically Modified T-cells in Treating Patients With Recurrent or Refractory Malignant Glioma	IL13Rα2	NCT02208362	Recruiting
Malignant Glioma, other advance solid tumors	1/2	10^*^	CAR-pNK Cell Immunotherapy in MUC1 Positive Relapsed or Refractory Solid Tumor	MUC1	NCT02839954	Recruiting
Glioma, other advance solid tumors	1/2	60^*^	A Clinical Research of CAR T Cells Targeting HER2 Positive Cancer	HER2	NCT02713984	Recruiting
Recurrent Glioblastoma	1	24^*^	Intracerebral EGFR-vIII CAR-T Cells for Recurrent GBM (INTERCEPT)	EGFRvIII	NCT03283631	Not yet open
Glioblastoma	1	48^*^	EGFRvIII CAR T Cells for Newly-Diagnosed GBM (ExCeL)	EGFRvIII	NCT02664363	Recruiting
Glioblastoma	1	20^*^	Pilot Study of Autologous Anti-EGFRvIII CAR T Cells in Recurrent Glioblastoma Multiforme	EGFRvIII	NCT02844062	Recruiting
Glioblastoma	1	16	CMV-specific Cytotoxic T Lymphocytes Expressing CAR Targeting HER2 in Patients With GBM (HERT-GBM)	HER2	NCT01109095	Ongoing, Not recruiting
Glioblastoma	1/2	20^*^	4SCAR-IgT Against Glioblastoma Multiform	EGFRvIII	NCT03170141	Open, invitation only
Glioblastoma	1	14^*^	T Cells Expressing HER2-specific Chimeric Antigen Receptors (CAR) for Patients With Glioblastoma (iCAR)	HER2	NCT02442297	Recruiting
Glioblastoma	1	20^*^	Pilot Study of Autologous Chimeric Switch Receptor Modified T Cells in Recurrent Glioblastoma Multiforme	PD-L1	NCT02937844	Recruiting

* *Means estimated sample size*.

## Challenges facing CART therapy in solid tumors

**Figure 4:**
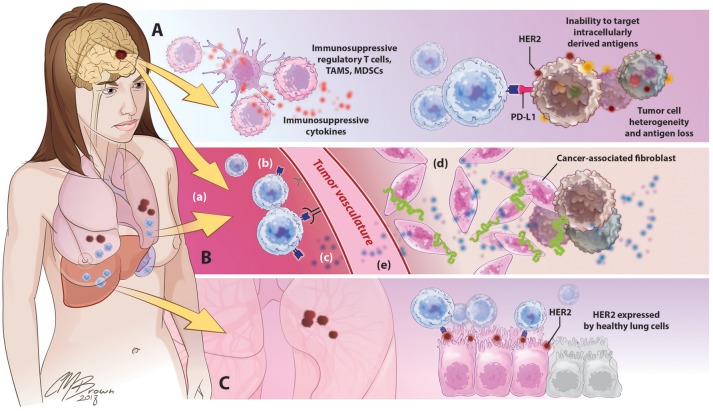
Barriers to successful CART immunotherapy in solid tumors. **(A)** Factors within the tumor microenvironment: Solid tumors contain an abundance of immunosuppressive leukocytes, immune checkpoint molecules, and suppressive cytokines. The tumor cells themselves are highly heterogeneous, preventing the identification of uniformly expressed targets for CAR design; antigen selection is further limited by an inability of CARTs to target intracellularly derived antigens. **(B)** Barriers to CART migration and entry into tumor sites: In contrast to the disseminated nature of hematologic cancers, solid tumors are often found in isolated locations that are difficult to access, like the brain. (a) following adoptive cancer, CARTs have been shown to preferentially accumulate in organs such as the lungs, liver, and spleen, with limited natural trafficking to tumor sites. (b) downregulated expression of ICAMs and other adhesion molecules on tumor vasculature limits lymphocyte extravasation, (c) reduced release of lymphocyte-attracting chemokines such as CXCL9-11 precludes CART homing to tumor sites, (d) in addition to supporting the growth and persistence of malignant cells, tumor-associated stroma provides both a physical and immunologic barrier to CART immunotherapy, (e) release of angiogenic factors such as VEGF promotes the develop of abnormal, tortuous, high-pressure vasculature that impedes lymphocyte entry.**(C)** Toxicity secondary to off-target effects: the use of overexpressed self-antigens as CAR targets introduces the risk of significant toxicity associated with CART identification and destruction of normal, healthy cells expressing these antigens.

For CAR T-cells to effectively eradicate solid tumors in human patients, they must be able to migrate to and infiltrate tumor tissues, proliferate and persist long enough to exert therapeutic effects, and identify and destroy only antigen-expressing cells within the TME. However, the ability for CART cells to these goals has thus far been hindered by a variety of factors unique to solid tumors, Figure 4.

### Selection of tumor-associated antigens

Perhaps the greatest challenge facing the successful application of CART technology to solid tumors in human patients is the selection of acceptable antigen targets. Solid tumors develop from the accumulation of a variety different mutations that promote unchecked cellular growth and proliferation, some of which result in the creation of tumor-exclusive epitopes that can be used to selectively identify malignant cells. However, rather than occurring linearly with the progression of a single homogenous line of cells expressing all accumulated mutations, tumorigenesis transpires through the simultaneous evolution and adaptation of individual malignant cells. As a result, solid tumors are comprised of highly molecularly heterogeneous subpopulations expressing a diverse, overlapping profile of unique TAAs. As a result, the current pool of potential antigens for CAR development in solid tumors is comprised of suboptimal targets that either lack uniformity of expression or tumor exclusivity, introducing additional concerns for efficacy and safety. In addition to precluding the identification of commonly and uniformly expressed targets for CAR development, this naturally-derived molecular heterogeneity both between patients and within individual tumors is difficult to reproduce in the human cell line-derived relatively homogeneous tumors utilized in preclinical experiments, limiting the ability of these studies to truly approximate human disease and predict patient responses to treatment (58).

To prevent inadvertent destruction of healthy tissues, CARTs are ideally designed to target antigens exclusively expressed by malignant cells. However, the vast majority of mutations producing novel epitopes in solid tumors occur through the processing of intracellular proteins, which are presented in the context of MHC molecules and therefore not accessible to traditional CARs. Due to a lack of known feasible tumor-exclusive antigens, many TAAs under current evaluation for CART immunotherapy are derived from overexpressed endogenous molecules, particularly those that promote tumor proliferation and persistence (i.e., growth factor receptors). Their disproportionately high degree of upregulation within tumors allows for preferential targeting of malignant cells. However, there are several important challenges and health risks associated with targeting overexpressed self-antigens that must be considered. Due to the natural development of tolerance to endogenous peptides, the majority of these overexpressed TAAs are poorly immunogenic. However, enhancing CART cytotoxicity against epitopes not restricted to malignant cells is limited by the danger of simultaneously promoting CAR recognition of target antigen expressed by healthy tissues. Toxicities secondary to unintentional destruction of non-cancerous cells has been observed to varying degrees following CART therapy targeting overexpressed self-antigens like CEA, a tumor-associated antigen that is also expressed in normal gastrointestinal epithelium. In a study of CEA-targeting CART immunotherapy for metastatic colorectal cancer, tumor regression was accompanied by severe inflammatory colitis in all treated patients, due to the destruction of healthy epithelial cells (59). Depending on the location of off-target antigen expression, the resulting toxicities may have life-threatening consequences, as occurred in a patient receiving CART therapy for refractory HER2-overexpressing metastatic colorectal cancer. This patient experienced fatal pulmonary toxicity shortly after CART infusion secondary to CAR recognition of low levels of HER2 on lung epithelium (60).

Identification of a tumor-exclusive antigen target feasible for CART immunotherapy is further complicated by the often inconsistent and incomplete degree of expression of unique tumor epitopes throughout the tissue. As a result, there likely exists a subpopulation of malignant cells lacking expression of any given target antigen that will therefore evade destruction by a corresponding CAR T-cell. In addition to reducing overall therapeutic efficacy this creates a selective pressure for the accumulation of cells with absent or mutated target antigen expression, introducing the potential for tumor recurrence through expansion of antigen-negative cell populations. This is particularly applicable when targeting epitopes derived from mutations that are not crucial to oncogenesis and tumor survival, as these structures can be readily modified or lost without compromising vital tumor functions. Indeed, therapy resistance coinciding with the emergence of antigen-loss variants is commonly observed in human patients with GBM and other solid tumors following treatment with CART immunotherapy (43, 45, 46, 61, 62).

### Lymphocyte trafficking

In contrast to the simplicity and ease of encountering of malignant cells in hematologic cancers, CARTs for solid tumors face the additional challenge of migrating to and infiltrating tumor sites. In humans and mice, CART persistence and intratumoral accumulation following systemic adoptive transfer is characteristically poor (63), with some studies showing initial trafficking to organs such as the lung, spleen, and liver, without any preferential accumulation in tumor sites (64). This limited trafficking may be multifactorial and impacted by both lymphocyte and tumor related influences, including settings of mismatch between tumor-expressed molecules and corresponding lymphocyte receptors (65) and the development of structural barriers surrounding tumor sites (66). With a reduced capacity to proliferate and relatively short overall persistence, CART immunotherapy in solid tumors becomes a “race against the clock,” as adoptively transferred cells have a limited amount of time to reach and destroy malignant cells, including those at sites distant to the bulk tumor.

Normal leukocyte migration and extravasation from the bloodstream is a multistep process that can be separated into four characteristic stages: (1) tethering/rolling, (2) activation, (3) adhesion/arrest, and (4) transmigration. Transient interactions between leukocyte carbohydrates and endothelial cell selectin receptors slows leukocyte velocity, causing them to roll along the endothelial wall in the direction of blood flow. These tethering interactions bring leukocytes in closer proximity to chemokines secreted by vascular endothelium, which bind leukocyte G-protein coupled receptors that activate the expression and adhesiveness of surface integrins, including lymphocyte function antigen-1 (LFA-1) and very late antigen (VLA)-4. Binding of these integrins to their ligands, like VCAM-1 and ICAM-1, present on tumor vasculature leads to a firm arrest of leukocyte motion. This stronger binding provides for transmigration into tumor sites (67).

In the setting of cancer, preferential migration of immune cells to tumor sites is mediated by tumor-secreted chemoattractants such as CCL2, CXCL9, CXCL10, and CXCL11 (65). An expression of corresponding receptors allows activated T-cells to respond to these factors, inducing migration in the direction of increasing chemokine concentration. This relationship between tumor-expressed chemoattractants and the profile of corresponding T-cell homing receptors is crucial in dictating a leukocyte's capacity to traffic to tumor sites (68). In a study of CTL trafficking in murine brain tumors, Okada et al. observed that CNS homing capacity was restricted to CTLs polarized toward a type 1, as compared to type 2, cytokine profile, demonstrating the importance of tumor tropism. These type 1 CTLs exhibited comparatively higher levels of endogenous VLA-4 and CXCR3, receptors that are crucial for CNS tropism (2). GBM and other highly vascular tumors characteristically overproduce vascular endothelial growth factor (VEGF), which, in addition to promoting angiogenesis, inhibits the secretion of lymphocyte-attracting chemokines like CXCL10 and CXCL11 (69) and downregulates tumor endothelial cell expression of adhesion molecules like ICAM-1 and ICAM-2, VCAM-1 and CD34 (70). Also abundant within the TME is the immunosuppressive cytokine TGF-β, which also downregulates endothelial cell expression of cellular adhesion molecules, thereby inhibiting T-cell transmigration (17).

Solid tumors also induce the development of structural modifications to the adjacent tissue that can prevent intratumoral lymphocyte accumulation. Many solid tumors are surrounded by a dense, peritumoral fibrous stroma that insulates nests of tumor cells from the surrounding environment (66). Forming the structural framework of this stroma are cancer-associated fibroblasts (CAFs), which play an important role in regulating tumor metabolism, growth, and persistence and mediate tumor invasion and metastasis through remodeling of the extracellular matrix (70, 71). Endogenous T-cells express heparanase, an ECM-degrading enzyme, which enhances their ability to penetrate this stroma; however, heparanase expression is often lost during *in vitro* culture, preventing CART from entering tumor sites (71). Abundantly secreted by tumor and stromal cells are CXCL-12, VEGF, and PDGF, which recruit bone marrow-derived endothelial cells and promote the development of abnormal, tortuous vessels leading to the development of abnormal, leaky vascular networks and elevated intratumoral interstitial pressures that resist lymphocyte infiltration (66).

### Tumor-induced immunosuppression

A major barrier to the efficacy of CART immunotherapy in solid tumors is a characteristic state of profound tumor-induced suppression of host antitumor immunity (72–74). The complex network of signals orchestrated by malignant and non-malignant cells of a tumor induces the development of an environment that is hostile to immune cell function and survival and favors tumor persistence. Through the elaboration of immunosuppressive cytokines and other soluble mediators, recruitment of inhibitory leukocytes, and activation of immune checkpoints, tumors create a microenvironment that inhibits effector cell activity and resists immune-mediated destruction. This multitude of immunosuppressive influences is responsible for the rapid loss of effector function observed upon CART entry into tumor sites (75).

Through surface expression of immunosuppressive molecules, solid tumors exploit endogenous regulatory pathways to directly inhibit T-cell effector functions. Among these immune checkpoints are the programmed cell death-1 (PD-1) and cytotoxic T-lymphocyte antigen-4 (CTLA-4) pathways. Activation of CTLA-4 receptors expressed by naïve T cells prevents their initial activation and stimulation of PD-1 on activated T-cells induces anergy, apoptosis, or development of immunosuppressive regulatory T-cells (Tregs). By upregulating PD-L1 and enhancing T-cell CTLA-4 and PD-1 expression, tumor cells are able to suppress the activity of incoming immune cells (76).

Present in high concentrations within the glioma microenvironment are immunoregulatory cytokines like transforming growth factor-β (TGF-β) and interleukin 10 (IL-10), which support the development of a type-2 polarized environment. In addition to suppressing MHC II molecule expression and antigen presenting cell maturation and function, these cytokines are potent inhibitors of T-cell differentiation, proliferation, and cytotoxicity (77, 78). Accumulating within gliomas and other solid tumors are inhibitory leukocytes like regulatory T-cells, myeloid-derived suppressor cells (MDSCs), and tumor-associated macrophages (TAMs), which are potent inhibitors of antitumor immunity (66). MDSCs constitute a diverse pool of immature myeloid cells that accumulate in settings of inflammation and malignancy. Through cell-cell contacts and the release of soluble mediators, MDSCs suppress the proliferation and function of antigen-specific CTLs and induce the development of Treg cells (79).

## Overcoming challenges and future directions

Although the application of CART immunotherapy to solid tumors continues to be faced with significant challenges, this therapeutic modality still holds the potential to make a significant impact in the field of solid tumors as it did for liquid malignancies.

### Antigen selection

Moving forward, the identification of commonly expressed antigen targets remains a constant challenge plaguing CART development for solid tumors. However, given the high degree of solid tumor heterogeneity existing across different patients, identification of such a broadly applicable antigen has been problematic. Despite the challenges facing antigen selection in solid tumors, CARs developed against a variety of TAAs have demonstrated encouraging antitumor efficacy and safety profiles in preliminary studies. One potential solution involves the study of individual intratumoral mutation profiles, which has revealed an exciting new category of possible antigen targets that may allow for the development of highly specific and effective personalized therapies (80, 81). Arising through somatic mutations within an individual tumor, these “neo-antigens” are inherently both tumor-specific and immunogenic. Endogenous T-cells naturally activated against these unique, patient-specific epitopes have been recovered from the tumors and peripheral blood of patients with a variety of solid cancers (82–84). Interestingly, patients with higher numbers of these immunogenic neo-antigens have been shown to possess a greater expansion of antigen-reactive CD8+ T-cells and elevated immune checkpoint expression, which may suggest synergy between CART immunotherapy and immune checkpoint blockade (85). Furthermore, the generation of these novel epitopes can be enhanced with other forms of immunotherapy known to trigger epitope spreading and activate antitumor immune responses, like dendritic cell-based vaccination or oncolytic virotherapy (86, 87). Aiding in the development of neo-antigen-based therapies, tumor exome and proteome analysis allows for a characterization of the full profile of neo-antigens within a single tumor and identification of epitopes that may be particularly immunogenic (88–90). Using this information, Castle et al. demonstrated the ability to induce T-cell immune responses against patient-specific neo-antigens via peptide vaccination against immunogenic epitopes (91). Given that the majority of these novel epitopes created within solid tumors are peptide-based antigens, CARTs have been developed in which the CAR ectodomain consists of a TCR-like construct that recognizes MHC-presented intracellular antigens (92). Indeed, transfection of T-cells with neo-antigen-specific receptors has been shown to be a viable option in preclinical studies (93, 94).

Targeting multiple TAAs has potential to enhance overall specificity for tumor cells, lessen the risk of off-target effects, and reduce the emergence of antigen loss variants and therapy-resistant tumors. After studying single-cell coexpression patterns of the TAAs HER2, IL-13Rα2, and EphA2 in primary GBM samples, Hegde et al. developed a mathematical model of antigen expression to predict the odds of complete tumor elimination with CART therapy. This model determined that a maximal expansion of CART therapeutic potential in all tumors can be achieved by co-targeting HER2 and IL-13Rα2, without any added advantage to targeting a third antigen. They subsequently developed bi-specific HER2 and IL-13Rα2-targeting CAR T-cells that exhibited enhanced antitumor activity and an ability to offset antigen escape in *in vitro* immunoassays and an orthotopic xenogeneic murine model (95). Tumor cell selectivity of bi-specific CAR T-cells can be further refined to only target cells expressing a specific antigen combination. This is achieved by physically separating CAR signaling domains between two distinct CARs specific for two different antigens, such that binding of both CARs to their individual target antigens is required to transmit an activating signal to induce T-cell cytotoxicity (96). This technique may be particularly effective against tumors that overexpress two antigens that are individually not exclusively expressed by malignant cells, but when coupled together, result in acceptable tumor specificity due to a lack of overlapping expression on non-target tissues.

Considering tumor-associated stroma is also important for therapy development. For example, in tumors with a high degree of antigen expression, CART immunotherapy has been shown to indirectly eradicate antigen loss variants, a process dependent on tumor stromal cell cross-presentation of TAAs (97). Further studies demonstrated that in settings of low antigen expression, local irradiation or chemotherapy was shown to cause sufficient release of antigen to sensitize stromal cells for T-cell mediated destruction, supporting introduction of CART immunotherapy into standard treatment regiments including radio and chemotherapy (98). Directly targeting antigens present on tumor-supporting structures such as tumor vasculature or associated stroma is also a promising option. CARTs directed against antigens preferentially expressed by tumor vasculature have been shown to directly disrupt these vascular networks leading to tumor regression in murine models of ovarian cancer and other vascular tumors (99). CARTs targeting fibroblast activation protein-α (FAP), a tumor stromal-associated antigen expressed by CAFs, delivered in combination with an EphA2-targeting CAR population was shown to control the tumor growth in an A549 lung cancer model more effectively than either agent alone (37).

### Lymphocyte trafficking

A variety of methods have been successfully employed to enhance CART trafficking to tumor sites including augmenting CART responsiveness to tumor-secreted cytokines, targeting tumor-associated stroma and the TME, and exploration of regional routes of delivery.

Fundamentally, T-cell migration and intratumoral accumulation will be facilitated by the utilization of T-cell populations expressing a profile of homing receptors allowing them to respond to the unique factors secreted by an individual tumor. To overcome tumor chemokine and lymphocyte receptor mismatches, CAR T-cells can be transduced with specific chemokine receptors known to dictate the appropriate tissue tropism (100, 101). For example, transfection of CARTs with a gene for CCRb2, the chemokine receptor for the T-cell chemoattractant CCL2, has been shown to enhance the migration and intratumoral accumulation of CARTs in xenograft models of multiple solid tumor types (33, 102). Brown et al. reported that glioma-derived CCL2/MCP-1 is ultimately responsible for the *in vivo* chemotaxis of adoptively transferred T-cells to glioma xenograft tumor sites (103), introducing promise for similar studies in glioma. CAR T-cells have been engineered to express heparanase, a modification that enhances intratumoral accumulation and overall antitumor activity (71). Combining multiple immunotherapeutic strategies to improve trafficking, one study evaluated CARTs delivered together with an oncolytic adenovirus engineered to express both a chemokine and T-cell growth factor. The combination of RANTES and IL-15 was shown to improve T-cell trafficking to tumor sites as well as create a favorable environment within the TME to enhance immune cell persistence (104).

In theory, any therapy that favorably modulates the TME or enhances the elaboration of relevant chemokines will enhance the migration of CARTs to tumor sites. In addition to mollifying the tumor-induced suppression of T-cell activity, immune checkpoint blockade with anti-PD-1 therapy augments lymphocyte trafficking in tumor-bearing mice by promoting the elaboration of the lymphocyte-attracting cytokines interferon-γ (IFN-γ) and CXCL10 (105). Similar effects are achieved with radiotherapy, which leads to IFN-γ-enhanced expression of adhesion molecules on tumor-associated vasculature (106). Anti-angiogenic therapy also promotes lymphocyte infiltration into tumor sites, and administration in combination with CART immunotherapy has been shown to be more therapeutically effective than treatment with either modality alone (107, 108). In addition to normalization of tumor vasculature, inhibition of VEGF signaling in a B16 melanoma model resulted in an 18-fold increase in intratumoral T-cell infiltration, which was associated with increased expression of CXCL10 and CXCL11 (69).

Proposed to circumnavigate many of the barriers limiting the trafficking of systemically delivered CARTs, regional CART administration may be a superior delivery strategy for CARTs in solid tumors. In contrast to the need for systemically delivered cells to traffic to tumor sites, intratumorally delivered cells have been shown largely to remain at the site of inoculation with minimal systemic absorption, which suggests that regionally delivered CARTs may carry a lower risk of off-target toxicities (63, 109). Several additional studies evaluating the efficacy of systemic and regionally delivered CART therapy have found regional delivery methods to result in superior T-cell persistence and overall therapeutic efficacy (32, 110). In one such comparison, regional intra-pleural delivery of mesothelin-targeting CAR T-cells resulted in earlier intratumoral accumulation, increased CD8+ T-cell proliferation and persistence, enhanced cytokine secretion, and improved overall therapeutic efficacy in an orthotopic model of human pleural malignancy. Adoptively transferred cells exhibited long-term persistence and protection against tumor rechallenge after 200 days (110). The application of such a regional delivery method may be especially advantageous in the setting of poorly accessible intracranial tumors like GBM that, nestled within a strongly immunosuppressive microenvironment, are significantly isolated from the systemic circulation. With respect to intracranial tumors, an *in vivo* study of breast cancer xenograft tumors with intracranial metastases was conducted to compare two regional CART delivery methods: intraventricular and a more localized, intracranial method. With both methods, functionally active, antigen-specific CD4+ and CD8+ T-cells were detected at tumor sites 1 week following administration. In this study, CARTs were rarely detectable by two weeks following administration Although equivalent antitumor activity was observed, some mice receiving the less localized intraventricular therapy exhibited more delayed therapeutic responses, demonstrating the need for adoptively transferred cells to traffic from the site of delivery (ventricle) to tumor sites (111). This delayed onset of action associated with T-cell trafficking makes the persistence of adoptively transferred cells of utmost importance. Specific to glioma model, intracerebral delivery of EGFRvIII-targeting CARTs has been shown to induce tumor regression in murine models of glioma (53). Several human clinical trials have employed regional CART delivery methods for the treatment of patients with progressive GBM. In one such phase I human clinical trial, following surgical tumor debulking, IL13Rα2-targeted CARTs were infused into the resection cavity via a Rickham catheter. Although this study did not assess the duration of CART persistence within the target tissue or at other locations, it proved that this route of administration was well tolerated and established feasibility for future evaluations (45). More recently, Brown et al. reported observations regarding two different regional CART delivery routes employed in the treatment of one patient with multifocal GBM. Transient anti-glioma responses were seen following initial intracranial CART delivery. After tumor recurrence, the patient was treated with intraventricular CART infusions and experienced robust tumor regression over the course of 7.5 months, though eventually succumbing to multifocal relapsed disease. Investigators evaluated CART persistence and cytokine levels in relation to intraventricular infusions. CAR T-cells were detected within the CSF for up to 1 week following infusion, with peak concentrations occurring after 2 days. Despite this lack of robust persistence, there was a significant induction of inflammatory cytokines, including IFN-γ, TNF-α, IL-12, IL-5, IL-6, IL-8, IL-10, CXCL9, CXCL10, CCR2, and IL-1Rα (46). Further studies will be needed to characterize and contrast the T-cell persistence and overall therapeutic efficacy associated with regional and systemically delivered CART therapies in the setting of GBM.

### CART cell persistence

The persistence of adoptively transferred T-cells is directly correlated with patient clinical responses and the degree of tumor regression (112). Although factors impacting CART persistence remain poorly understood, there have been several key advances in this field that have shown promise for optimizing persistence and associated therapeutic efficacy.

The selection of different costimulatory motifs can confer unique properties in terms of cellular metabolism, cytokine secretion, cytotoxicity, proliferation, persistence, and memory cell generation. Costimulation with 4-1BB derived moieties promotes the generation of long-lived central memory cells with enhanced mitochondrial biogenesis and fatty acid oxidation. Signaling through a different pathway, CD28 leads to the differentiation of shorter-lived effector memory cells and metabolic changes leading to increased reliance on aerobic glycolysis for proliferative energy (113). A diverse combination of different subpopulations of CARTs possessing the unique strengths and weaknesses of differential costimulation may provide the most effective overall antitumor effect.

In addition to structural CAR modifications, CART persistence is classically enhanced by pretreatment lymphodepletion with cyclophosphamide or fludarabine (114). Lymphodepletion is thought to support T-cell engraftment by depleting populations of suppressive host lymphocytes, like Tregs, and eliminating competition for cytokines like IL-7, IL-15, and IL-21, which promote T-cell activation and expansion (115). This effect can be further augmented with the administration of supportive cytokines (116). The most widely studied is IL-2, however in addition to supporting CTL persistence and function, IL-2 also promotes the expansion of immunosuppressive regulatory T-cells (117). In contrast, IL-15 has been shown to preferentially promote cytotoxic T-cell persistence and function even in the presence of Tregs (118). Persistence and cytotoxicity are also influenced by the composition of the transferred CART lymphocyte population (119), with infusions containing higher numbers of CD4+ and memory T-cells exhibiting longer overall longevity (57).

### Overcoming tumor-induced immunosuppression

The clinical success of CART immunotherapy depends upon the ultimate ability of tumor-infiltrating CTLs to retain their ability to identify and destroy malignant cells within this suppressive microenvironment. The immunosuppressive effects of the TME can be addressed through a variety of strategies that enhance the effector functions of adoptively transferred cells and/or directly counteract mechanisms of tumor-induced immunosuppression. In addition to exogenous administration of adjunctive therapies, CAR T-cells themselves can be genetically engineered to secrete immune-modulating compounds or to reduce their susceptibility to tumor-induced immunosuppression.

The success of CART immunotherapy can also be improved by strategies that focus on altering the anti-inflammatory and immunosuppressive nature of the TME by armoring them with immune modulating cytokines. CARTs engineered to express IL-12 upon antigen binding release this pro-inflammatory cytokine into the surrounding milieu, which functions to promote an environment favoring antitumor immunity. By counteracting the immunosuppressive nature of the TME, IL-12 lessens the recruitment and suppressive influences of immature dendritic cells, TAMs, Tregs, and MDSCs, and recruits additional innate immune cells that may be able to destroy tumor cells that are not visible to CARs (120). IL-12-expressing CARTs also exhibit improved antitumor efficacy and have been shown to be intrinsically resistant to Treg-mediated immunosuppression (121). Overall, delivery of pro-inflammatory cytokines like IL-12 has been shown to counter the immunosuppressive nature of the local microenvironment by promoting the polarization T-cell responses toward the T helper-1 phenotype, enhancing IFN-γ release, augmenting granzyme and perforin production, suppressing angiogenesis, and reducing the population of infiltrating MDSCs (122, 123). Antibody-mediated therapy to deplete suppressor cells, including MDSCs and Tregs, has also been shown to further reduce tumor burden (32). Alternatively, CARTs can be modified to resist immunosuppressive influences within the TME. Present in high amounts within tumor sites, TGF-β has a profound inhibitory influence on the activity of immune effector cells. CTLs transfected with a dominant negative TGF-β receptor are rendered insensitive to TGF-β-mediated suppression and were shown to have enhanced antitumor activity as compared to unmodified CTLs (124). Engineering CAR T-cells to be resistant to the multitude of immunosuppressive influences within the TME is a constant goal in the development of more sophisticated therapies.

Given the complexity of immune interactions within solid tumors and their importance in disease outcome, there inherently exists significant synergistic potential in combination treatment with CARTs and other forms of immunotherapy, such as dendritic cell-based vaccination, oncolytic virotherapy, and immune checkpoint blockade (87, 125). Supporting this assertion, simultaneous PD pathway blockade has been shown to improve the cytotoxic activity of adoptively transferred CAR T-cells, reduce the presence of MDSCs, and enhance tumor growth inhibition in murine models (126). Overall, in addition to attracting CARTs to tumor sites, the resulting enhanced secretion of IFN-γ and CXCL10 creates a pro-inflammatory TME favoring the development of antitumor immunity (105). Immune checkpoint blockade with anti-PD pathway antibodies can be achieved either through the exogenous coadministration or via CART secretion. Anti-PD-L1 antibodies secreted by CARTs have been shown to induce an efficient, localized PD blockade that promoted T-cell cytotoxicity, NK cell recruitment, and tumor growth control in a murine model of colorectal carcinoma (127). An alternative, potentially more efficacious, strategy to blocking immune checkpoint signaling is exchanging the inhibitory signal transmitted by checkpoint activation for a stimulatory one. T-cells can also be engineered to express decoy receptors containing an extracellular domain similar to CTLA-4 or PD-1 that transmits an activating signal. CARTs expressing a PD-1-CD28 switch receptor were shown to be more resistant to tumor-induced immunosuppression, and in a solid tumor mouse model resulted in enhanced TIL infiltration and tumor regression as compared to treatment with CART therapy or PD-1 blockade alone (128).

## Conclusion

In conclusion, CART therapy has significant unrealized potential to revolutionize the treatment of solid tumors, as it has already done for hematologic cancers. A better understanding of factors leading to the success of CART immunotherapy in these liquid cancers and an analysis of those limiting the applicability of CART therapy to solid tumors will be necessary to move this field forward. Strategies that address the unique aspects of solid tumor biology like molecular heterogeneity, isolated tumor location, and associated immunosuppression must be thoroughly studied to extend the use of CART immunotherapy to these tumors. Further studies involving optimization of *ex vivo* culture conditions, genetic manipulation of CAR and CART structure, and investigation of combination therapies are necessary to provide a lasting solution.

## Author contributions

AF and MH contributed to literature review and writing. MD contributed to article conception, writing, and overall supervision.

### Conflict of interest statement

The authors declare that the research was conducted in the absence of any commercial or financial relationships that could be construed as a potential conflict of interest.
